# Novel epigenetic molecular therapies for imprinting disorders

**DOI:** 10.1038/s41380-023-02208-7

**Published:** 2023-08-25

**Authors:** Sung Eun Wang, Yong-hui Jiang

**Affiliations:** 1 Department of Genetics, Yale University School of Medicine, 333 Cedar street, New Haven, CT 06520 USA; 2 Department of Neuroscience, Yale University School of Medicine, 333 Cedar street, New Haven, CT 06520 USA; 3 Department of Pediatrics, Yale University School of Medicine, 333 Cedar street, New Haven, CT 06520 USA

**Keywords:** Genetics, Diseases

## Abstract

Genomic imprinting disorders are caused by the disruption of genomic imprinting processes leading to a deficit or increase of an active allele. Their unique molecular mechanisms underlying imprinted genes offer an opportunity to investigate epigenetic-based therapy for reactivation of an inactive allele or reduction of an active allele. Current treatments are based on managing symptoms, not targeting the molecular mechanisms underlying imprinting disorders. Here, we highlight molecular approaches of therapeutic candidates in preclinical and clinical studies for individual imprinting disorders. These include the significant progress of discovery and testing of small molecules, antisense oligonucleotides, and CRISPR mediated genome editing approaches as new therapeutic strategies. We discuss the significant challenges of translating these promising therapies from the preclinical stage to the clinic, especially for genome editing based approaches.

## Introduction

Genomic imprinting is a special form of epigenetic regulation, resulting in monoallelic gene expression depending on parent-of-origin. Genomic imprinting was first described in the early 1980s in mice from the elegant pronuclear transfer experiments by Surani and Solter [1, 2]. They showed that the contribution of the parental genomes to offspring genomic transcriptions were nonequivalent. Insulin-like growth factor 2 receptor (*Igf2r*) was the first imprinted gene discovered in mice in 1991 [3]. In humans, ~130 genes have been reported as imprinted genes and additional ~120 genes are predicted or provisioned (geneimprint.com). A number of imprinted genes are essential for normal embryonic development and neurodevelopment [4–6]. Several distinct features are associated with imprinted gene regulation [7–10]. First, the imprinted genes are frequently clustered and under a coordinated epigenetic regulation. Two major clusters of imprinted genes are in the chromosome 11p15.5 and 15q11-q13 regions. Second, the imprinted genes are frequently associated with allelic specific epigenetic modifications of DNA methylation, post-translational histone modifications, and chromatin structure [11–14]. Third, a significant number of imprinted genes are noncoding RNAs [15, 16]. Fourth, the antisense and long noncoding RNAs (lncRNAs) are often implicated in regulating imprinted expression [16–20]. These features play a significant role in establishing and regulating imprinting mechanisms and also present as targets for the development of molecular based therapies [21, 22].

The disruption of imprinting processes during gametogenesis and the expression of imprinted genes causes significant developmental defects and diseases in humans referred to as genomic imprinting disorders [6, 9, 23]. Epigenome wide association studies and genome wide differentially methylated region analyses have found aberrant epigenetic changes in imprinted and non-imprinted loci. These changes are either germline or somatic origin that could be associated with genetic variants or environmental insults such as nutritional factors and endocrine-disrupting chemicals etc [9]. Somatic origin changes are frequently cell, tissue type, and developmental stage specific [14, 24–26]. These changes are referred to as “Epimutations” collectively [9, 27]. The causal role of epimutations in cancer susceptibility has been better characterized over the last two decades but remain to be established whether these changes are implicated in non-cancer related diseases [28–30].

Prader–Willi (PWS) and Angelman syndrome (AS) are the first examples of genomic imprinting disorders described in the late 1980s [31–34]. Currently, there are 15 genomic imprinting disorders described in humans (Table 1). The clinical features reported in imprinting disorders span many organ systems and functional domains and are usually debilitating and lifelong conditions. Neurodevelopmental and neuropsychiatric presentations such as intellectual disability (ID), autism spectrum disorder (ASD), and other psychiatric presentations are notable features associated with the majority of imprinting disorders [35]. For example, psychosis is reported in 10–20% of adult PWS patients and more common in cases resulting from maternal uniparental disomy (UPD) [36].

**Table 1:** Molecular basis of imprinting disorders.

Imprinting disorder [OMIM]	Chromosome/Imprinted gene	Molecular defects	Key clinical features	Current treatment
Diabetes mellitus, transient neonatal,1 [601410]	6q24/*PLAGL1*, *HYMAI*	UPD (chr6) pat, paternal duplications, methylation defects on *PLAGL1* TSS (caused by mutation in *ZFP57* gene at chr6p22)	Intrauterine growth retardation, neonatal diabetes mellitus, hyperglycemia, macroglossia	Insulin
Silver-Russell syndrome 1 [180860] 2 [618905] 3 [616489]	11p15.5/*H19, IGF2* 7p13-q32/*C7ORF10* 11p15.5/*IGF2*	UPD (chr11p15) mat, chr11p15 CNVs, UPD (chr7) mat, methylation defects on *H19* and *IGF2* genes	Pre- and postnatal growth deficiency, asymmetry, broad forehead, facial dysmorphism, macrocephaly, gastrointestinal symptoms	Growth hormone
Birk–Barel syndrome [612292]	8q24.3/*KCNK9*	*KCNK9* missense mutation on maternal gene	Developmental delay, central hypotonia, facial dysmorphism, intellectual disability,	Symptomatic care
Beckwith–Wiedemann syndrome [130650]	11p15.5/*H19, IGF2*	UPD (chr11p15) pat, hypermethylation on *H19* and *IGF2* genes imprinting control region (ICR1)	Hypoglycemia, hyperinsulinism, Macroglossia, overgrowth, abdominal abnormalities	Surgery to treat cases with omphalocele or umbilical hernia
Kagami–Ogata syndrome [608149]	14q32/ *DLK1,MEG3, RTL1, MEG8, miRNAs, SNORDs, DIO3, RTL1as*	UPD (chr14) pat, 14q32 mat deletion, hypermethylation on *MEG3–DLK1* region	Polyhydramnios, macrosomia, placentomegaly, hypotonia, developmental delay, abdominal wall defects, cardiac/thoracic abnormalities	Growth hormone
Temple syndrome [616222]	14q32/ *DLK1,MEG3, RTL1, MEG8*, miRNAs, *SNORDs, DIO3, RTL1as*	UPD (chr14) mat, 14q32 pat deletion, hypomethylation on *MEG3–DLK1* region	Postnatal short stature, hypotonia, developmental delay, small hands and feet, intellectual disability	Growth hormone
Prader–Willi syndrome [176270]	15q11.2-q13/*MRKN3, MAGEL2, NDN, PWRN4, PWRN3, SNURF-SNRPN, SNORDs* IPW	chr15q11.2-q13 pat deletion, UPD (chr13) mat, imprinting defect, deletion of paternal SNORD116	Hypotonia, developmental delay, intellectual disability, hyperphagia, hypogonadism, diabetes type II	Growth hormone
Angelman syndrome [105830]	15q11.2-q13/*UBE3A*	chr15q11.2-q13 mat deletion, UPD (chr13) pat, imprinting defect, *UBE3A* point mutation on maternal allele	Unmotivated laughing, ataxia, microcephaly, developmental delay, seizures, hyperreflexia	Anti-epileptic drug
Central precocious puberty 2 [615356]	15q11.2/*MKRN3*	*MKRN3* point mutations on the paternal allele	Gonadotropin-dependent precocious puberty	GnRH analog therapy
Schaaf–Yang syndrome [615547]	15q11.2/*MAGEL2*	*MAGEL2* point mutations on the paternal allele	Psychomotor developmental delay, hypotonia, behavioral abnormalities, intellectual disability	Growth hormone, anti-psychotic medication, non-invasive ventilation
Pseudohypopara-thyroidism1A (PHP1A) [103580] / PHP1C [612462]	20q13.32/*GNAS*	Inactivating mutation on maternal allele of *GNAS*	Albright hereditary osteodystrophy, resistance to parathyroid / other hormones, obesity, cognitive impairment	Growth hormone, Vitamin D
Pseudohypopara-thyroidism1B (PHP1B) [603233]	20q13.32/*STX16, GNASAS1, GNAS*	Imprinting defect on maternal allele of *GNAS*, 20q13 mat deletion, UPD (chr20) pat	Albright hereditary osteodystrophy, resistance to parathyroid / other hormones, cognitive impairment	Calcium, Vitamin D
Pseudopseudohypo-parathyroidism (PPHP) [612463]	20q13.32/*GNAS*	Inactivating mutation on paternal allele of *GNAS*	Albright hereditary osteodystrophy, mental retardation	Calcium, Vitamin D
Progressive osseous heteroplasia [166350]	20q13.32/*GNAS*	Inactivating mutation on paternal allele of *GNAS*	Heterotopic ossification	Bis-phosphonate pamidronate
Mulchandani–Bhoj–Conlin syndrome [617352]	20q11-q13/*GNAS*	UPD (chr20) mat	Prenatal growth restriction, severe short stature, proportional head circumference, profound feeding difficulty	Growth hormone

Recent large scale genomic studies have uncovered a list of genes that encode proteins of epigenetic machinery implicated in neurodevelopmental disorders (NDD) specifically for ASD [37–40]. For example, allelic specific modifications such as DNA methylation and histones are frequently associated with imprinted genes [41, 42]. Mutations in genes encoding DNA methyltransferase (*DNMT3A*), DNA demethyltransferase (*TET3*), H1 linker histone, histone modifying enzymes, and chromatin remodelers (*KDM5B*, *EZH2*, *EHMT1*, *CTCF*, etc.) have been implicated in ASD and NDD [40, 43–45]. It remains to be investigated whether deficiencies of these proteins indirectly affect the genomic imprinting process and their involvement in imprinting disorders.

**Fig. 1:**
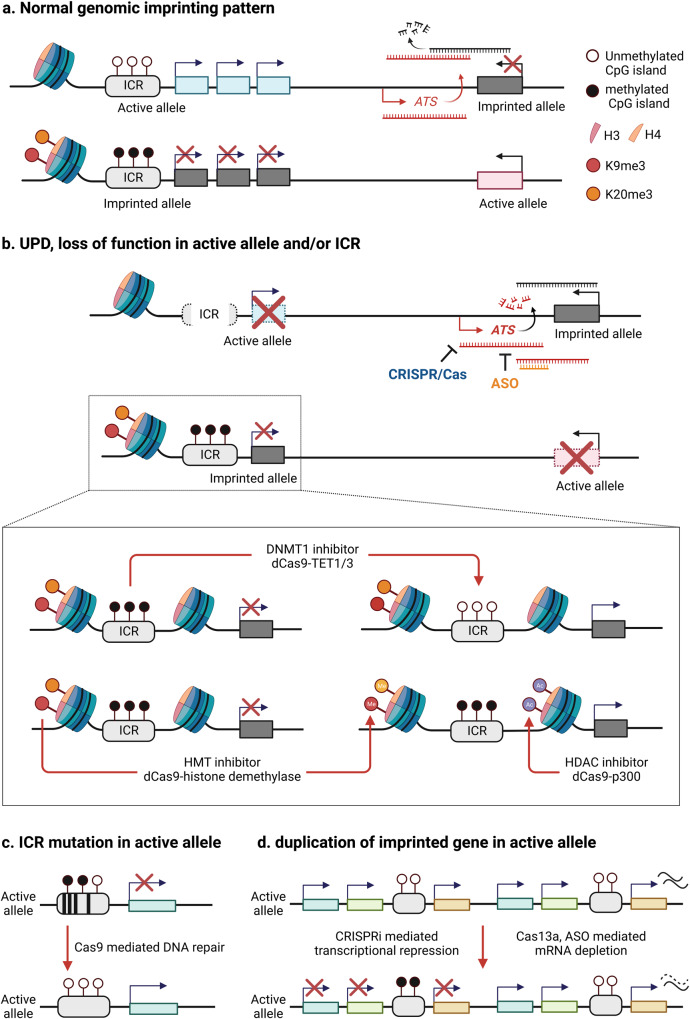
Epigenetic-based treatment strategies for imprinting disorders. Schematic diagram describes epigenetic-based therapeutic targets including DNA methyltransferase and histone modifying enzymes for imprinting disorders. **a** Normal genomic imprinting pattern shows parental origin specific allele silencing. **b** Uniparental disomy (UPD), deletion or mutation of active allele/imprinting control region (ICR) causes deficiency of normal gene expression. CRISPR/dCas9 or small molecule-mediated reactivation of silenced (imprinted) genes is applicable to recover normal gene expression. **c** Allele specific CRISPR/Cas9-mediated genome editing can be a tool for correction of ICR mutation resulting in an imprinting defect or epimutation. Designing allele specific gRNA is required to do single nucleotide polymorphism (SNP) analysis to distinguish mat/pat chromosome. **d** Two copies of imprinted gene in active allele can be repressed by CRISPRi (ex. dCas9-KRAB, DNMT1), CRISPR/Cas13 and ASO mediated mRNA depletion (ASO antisense oligonucleotides, ATS antisense transcripts, DNMT1 DNA methyltransferase 1, HMT histone methyltransferase, HDAC Histone deacetylase). Created with BioRender.com.

Like most other genetic disorders, there are no effective molecular treatments for imprinting disorders. Current treatments for imprinting disorders are symptom-based interventions and often ineffective. The molecular mechanism underlying imprinting disorders is primarily due to loss of active alleles or duplication of repressed or inactive alleles via different genetic mechanisms [9, 12, 46]. In rare cases, duplication of an active allele can also cause an imprinting disorder [11, 47, 48]. Common genetic defects include a copy number variant (CNV) resulting in loss (e.g. chromosome deletion), DNA sequence variants in an active allele resulting in loss of function of an imprinted gene, and uniparental disomy (UPD) of the repressed allele for an imprinted gene. More rare genetic etiologies include microdeletions or epimutations in the imprinting center/control region (ICR), a regulatory element that controls allele-specific expression of genes in an imprinting domain. Allele-specific epigenetic markers such as DNA methylation and histone modifications are frequently reported in the ICR region [9]. Repressed epigenetic markers are usually associated with repressed alleles and vice versa. These unique molecular features render an exciting opportunity to explore epigenetic-based therapy for imprinting disorders (Fig. 1).

Epigenetic-based therapy has been investigated extensively in cancers and mostly used the pharmacological approach [49–51]. The knowledge learned from cancer studies provides valuable insights for the mechanism and paves a convenient pathway for preclinical imprinting disorder studies in terms of the requirement of regulatory processes for US Food and Drug Administration (FDA) approval. This review describes the exciting development of potential epigenetic therapies targeting imprinting disorders by manipulating chromatin remodeling factors at the level of histone, DNA, and RNA by different molecular approaches. We will highlight CRISPR/Cas9, Cas13 or dCas9 mediated epigenome editing as potential therapies for imprinting disorders using AS and PWS as prime examples.

## Scientific premise for epigenetic-based therapy

The molecular basis of epigenetic based therapy for imprinting disorders is to unsilence/reactivate the expression of disease-causing genes from the repressed allele in imprinted loci via pharmacological or molecular genetic manipulation. The basic concept of epigenetic-based therapy has been intensely studied in cancers for more than two decades mostly by pharmacological approach [49–51]. Many drugs have been developed or tested that could affect epigenetic modifications at DNA and histone components. Recently, molecular approaches such as antisense oligonucleotide (ASO) [52], shRNA, and CRISPR/Cas9 mediated gene editing have emerged as major interests. The difference between cancers and imprinting disorders is that epigenetic-based therapy for imprinting disorders is allele-specific which is not the case in cancers. Cancer treatments are typically organ or cell-type specific given the nature of somatic mutations but imprinting disorders are mostly of germline origin. In recent years, studies exploring epigenetic-based therapy for imprinting disorders have gained interest and momentum because of the FDA approval of launching several clinical trials using epigenetic-based approach. The basic scientific premise and approach for epigenetic based therapy is diagrammed in Fig. 1 and discussed in detail below. Conceptually, the design of epigenetic-based therapy may target the individual imprinted gene or the imprinting center region that is expected to change the expression of a cluster of imprinted genes within the imprinting domain.

## Small molecules for epigenetic modifications

Epigenetic modifications can be achieved by small molecules or other pharmacological interventions altering the function of epigenetic machinery directly associated with transcriptional regulation of imprinted genes. Histone modifying enzymes are common targets for development of novel therapies for human diseases, primarily for cancers. Many small molecules targeting histone deacetylases (HDACs) have been approved by the FDA and are in clinical trials and or used as therapies for cancers and other diseases [53]. These include vorinostat, belinostat, romidepsin, tucidinostat and panobinostat. Significant side effects are reported in previous studies associated with these drugs [54]. Vorinostat is currently in trials for Alzheimer’s disease (NCT03056495) as well as epilepsy (NCT03894826). Valproic acid (VPA, Depakene) is a widely prescribed drug to treat epilepsy in children and used as a mood stabilizer in adults. It has been shown experimentally that VPA possesses HDAC activity, although it is unknown whether this is the mechanism underlying its clinical efficacy. Recent evidence suggests that epigenetic aberrations do play a role in epileptogenesis and that HDAC inhibition may be an attractive mechanism to target for treatment and prevention of epilepsy [55, 56]. Similar to VPA, preclinical studies of Phenylbutyrate (PBA) suggest a possible role as a HDAC inhibitor, and is currently FDA approved for treatment of urea cycle disorders in the form of sodium (Buphenyl) and glycerol (Ravicti) salts. Recently, PBA has been explored in phase 1 and 2 clinical trials for treatment of a range of neurodegenerative diseases, including Alzheimer’s disease, amyotrophic lateral sclerosis [57, 58], and Parkinson’s disease (NCT02046434).

To systemically identify new epigenetic drugs, high content small molecule screenings have been carried out to examine the feasibility of reactivating the repressed imprinted genes using proper markers in cell-based assays [22]. Screenings of small molecule libraries have been performed to investigate potential therapeutic targets for imprinting disorders using mouse models. Using the primary neurons from a reporter mouse model that carries YFP fused to AS *Ube3a* gene, Philpot and Roth groups have screened >3000 small molecules [59]. This screen identifiies a class of topoisomerase inhibitor that unsilences the expression of AS *Ube3a* gene from the repressed paternal chromosome in neurons both in vitro and in vivo. One of these topoisomerase inhibitors is topotecan, a FDA approved drug for treatment of metastatic cancers. Intervention with topotecan in the AS *Ube3a* maternal deficiency mouse model rescues the neurobehavioral phenotypes. The significant toxicity associated with topotecan has precluded it from being considered further in human AS. In a mouse model for PWS, EHMT2/G9a inhibitors are found as effective small molecules to unsilence the expression of normal repressed PWS candidate genes from the maternal chromosome 15q11-q13 region in both human PWS derived cells and PWS mouse model [60]. The biological effect of EHMT2/G9a inhibitors is to reduce the H3K9me2 level. Interestingly, treatment with an EHMT2/G9a inhibitor reduces the level of H3K9me2 in the PWS-ICR without changing its DNA methylation. While the treatment of EHMT2/G9a inhibitor is well tolerated in rodents at different ages, it remains to be investigated whether these EHMT2/G9a inhibitors are safe in humans. In a mouse model for Birk–Barel syndrome, HDAC inhibitor reactivates the paternal silenced *Kcnk9* allele with an increase of H3K27 acetylation in *Kcnk9* promoter and intronic regions [61]. This inhibitor does not change the allele-specific DNA methylation of *Peg13*, a differentially methylated region located upstream of *Kcnk9* gene [61]. These similar approaches are applicable for identification of effective small molecules to modulate the expression of repressed genes in other imprinting disorders. The biggest challenge of translating these molecules into treatment of human imprinting disorders is the specificity and toxicity of these small molecules. In contrast to drugs targeting terminal cancers using small molecules, the safety threshold of targeting patients with imprinting disorders should be higher.

## Antisense oligonucleotide (ASO) mediated reactivation of imprinted genes

ASOs are short, synthetic, single-stranded oligodeoxynucleotides that bind to target pre-mRNAs [62]. Chemically modified ASOs are internalized by active transport or passive diffusion for nucleus entry [63]. Depending on their design and chemical modification, ASOs regulate mRNA levels or alternative splicing through different mechanisms, resulting in changes of mRNA and protein expression [62].

As a therapeutic strategy, ASOs have been introduced and extensively tested for over two decades to improve their activity in clinical trials [64]. With technical advances in their efficacy, about 10 ASO-mediated therapies have been approved by the FDA for genetic and non-genetic disorders [65–67]. The most notable success is the ASO treatment of spinal muscular atrophy [68]. These FDA approved ASO based treatments for genetic disorders have paved the way to obtain FDA approval for imprinting disorders. ASO based treatment has emerged as a promising molecular treatment for AS. The AS *UBE3A* gene is subject to brain specific imprinting [69, 70]. The *UBE3A* gene is exclusively expressed from the maternal chromosome in neurons [71]. The exact mechanism underlying the brain and neuron specific imprinting for the AS *UBE3A* gene remains fully characterized. It has been demonstrated in rodent models that the maternal and neuron specific expression of *Ube3a* is mediated by the expression of a paternally expressed long non-coding antisense RNA to *Ube3a* (*Ube3a-ATS*) [18, 72]. Inhibition or inactivation of *Ube3a*-ATS by ASO and CRISPR based gene editing at the DNA and RNA levels unsilence the expression of sense *Ube3a* in brains [73–76]. The reactivation of *Ube3a* by ASO is capable of rescuing the neurobehavioral and neurophysiological impairments in AS maternal *Ube3a* deficiency mouse model [73, 77–79]. These findings led to a successful approval of investigational new device (IND) for using ASO in treating human AS. In 2020, the FDA approved the first ever phase 1 trial for using ASO via intrathecal injection in AS. Currently, there are 4 active phase 1/2a clinical trials using different ASO designs sponsored by IONIS (NCT05127226), Hoffmann-La Roche (NCT04428281), and Ultragenyx (NCT04259281) in the US and other countries. While the phase 1/2a trials are not primarily designed to assess clinical efficacy, the assessments from these trials revealed encouraging positive signals in multiple behavioral domains [52].

## CRISPR/Cas9 genome editing

The studies of ASOs provide a proof of concept to support molecular therapy by manipulating *UBE3A-ATS*. However, due to the transient nature of ASO treatment after entry into cells, repeated intrathecal injections are necessary to maintain clinical efficacy if it eventually becomes a standard therapy. The requirement of sedation for intrathecal administration of ASOs poses significant medical and psychosocial stress to AS children and families. CRISPR/Cas9 gene editing offers an attractive alternative to ASO. Two recent studies have demonstrated that Cas9 mediated gene editing can inactivate the expression of *Ube3a-ATS* and reactivate the expression of *Ube3a* from the paternal chromosome in vitro and in vivo using an adeno-associated virus (AAV) or lentivirus delivery method [74, 75]. A single intrathecal delivery could achieve long term and probably permanent molecular efficacy. Similar to ASOs, the Cas9 editing of *Ube3a-ATS* rescues the neurobehavioral phenotypes in the AS *Ube3a* maternal deficiency mouse model. These studies provide initial evidence supporting the feasibility of using Cas9 editing to treat AS. Like CRISPR/Cas9 mediated gene editing in other genetic diseases, safety concerns related to virus delivery methods and potential off-target effects due to Cas9 editing remain to be evaluated thoroughly before moving to human trials. Conceptually, an unbiased CRISPR based screening could be designed to screen for a genetic locus that could unsilence imprinted genes and lead to the development of a new treatment. This has not been reported in literature so far.

## CRISPR mediated RNA editing

Non-coding RNAs are frequently associated with imprinted gene clusters and strongly implicated in the regulation of imprinted clusters [16, 80–82]. The CRISPR/Cas13 system is an effective tool for RNA editing [83], where Cas13 controls RNA without permanent change of DNA sequence in gene bodies. In contrast, Cas9 has technical limitations including low editing efficiency, higher probability of inducing off-target events and oversized AAV packaging [84]. Cas13 modulates RNA readout through various modifications such as methylation, demethylation, and A-I/C-U editing. Cas13a with gRNA has proven RNA-guided RNA knockdown [85–87]. Conceptually, this tool can be used to target specific lncRNAs controlling imprinted domains or reactivate imprinted genes. dCas13b-ADAR2 has a function of A to I RNA base editing [83, 84], which has the important role of correcting pathogenic mutations at the RNA level. dCas13-METTL3/14 as a N6-adenosine-methyltransferase (A to m^6^A), increases RNA stability and translation efficiency, leading to enhancement of gene expression [84]. CRISPR-Cas13d variants such as dCasRx is the smallest form targeting RNA, and is able to be packaged into lentivirus for improved delivery efficiency to primary cells [88]. As a m^6^A demethylase, dCasRx-ALKBH5 shows bidirectional modulation depending on targeting mRNA [89]. The technical challenge for the translational potential of Cas13 may be related to targeting deliverance of the editing tool to specified organs and cell types. Recently, the use of Cas13 based editing of *UBE3A-ATS* has been reported to reactivate the expression of *UBE3A* from the paternal chromosome and rescue some neurobehaviors in mice [76]. However, like ASOs, the transient effect of Cas13 RNA editing is expected to require repeated interventions to maintain efficacy.

## CRISPR/dCas9 mediated epigenome editing

CRISPR-based epigenome editing technologies have been developed to enable manipulation of the epigenome and regulate expression of targeted genes [90–94]. For the design of epigenome editing, a catalytically inactive mutant form of Cas9 (dCas9) without endonuclease activity still binds to target DNA sequence that matches guide RNA. dCas9 fused with an effector domain has emerged as a popular approach to target a specific locus with specific modifications. The fusion constructs of dCas9 include various catalytic domains of epigenetic modifying enzymes and chromatin remodelers that have been demonstrated to result in transcriptional activation or repression for a targeted gene. Recently, Cas13 has been shown to improve the dCas9 platform targeting DNA and histones or recruiting other transcriptional factors [83, 86]. Chemically modified guide RNA could also improve the transcript knockdown efficiency with CRISPR/Cas13 as a form of ribonucleoprotein (RNP) complex [76, 95]. Conceptually, epigenome editing is applicable to target allele-specific DNA and histone modifications associated with repressed alleles and reactivate the expression of imprinted genes [96].

## DNA methylation and demethylation modification and editing for imprinted genes

5-methylcytosine (5mC) in CG dinucleotides (CpG) plays a critical role in imprinting establishment during development [97–99]. CpG islands are mainly located in transcriptional regulatory elements such as promoter, inhibitor, and enhancer regions [100]. In the imprinting domain, allelic methylation of CpG dinucleotides is frequently identified in the imprinting center. DNA methyltransferase (DNMT), an epigenetic writer, has a primary role in establishing and maintaining DNA methylation, resulting in subsequent recruitment of repressor complex for gene silencing in general. dCas9 fused with mammalian DNA methyltransferase of DNMT1, DNMT3A, DNMT3B, and DNMT3L and prokaryotic DNA methyltransferase MQ1 displayed de novo methylation with inhibition of transcription in preclinical studies [101–105]. This tool can be applied to imprinting disorders caused by duplication of active allele, such as transient neonatal diabetes mellitus type 1 (paternal duplication of 6q24) and Beckwith–Wiedemann syndrome (paternal duplication of 11q15.5) [106].

As an enzymatic eraser, the ten-eleven translocation dioxygenase family of genes (TET1-3) encodes enzymes that oxidize 5mC to 5-hydroxymethylcytosine (5hmC) leading to active DNA demethylation and increase of gene transcription in general. dCas9-TET1/TET3 targeting to methylated CpG island contributes to specific gene activation in various disease models [92, 107–109]. The dCas9-TET1 fusion construct can demethylate the methylated 5mC associated CGG repeat expansion of *FMR1* (Fragile X Syndrome) in cells and in vivo [102], the CpG island of the maternally imprinted *Snrpn* gene in a rodent model [92], and the *MECP2* promoter for its reactivation from inactivated Xi chromosome in Rett syndrome human embryonic stem cells and derived neurons [110]. With the same principle, the dCas9-TET fusion protein can be used to reactivate imprinted genes that have been shown to be associated with methylated CpG islands in regulatory regions.

## Histone modification editing

Manipulating posttranslational modifications of histones in in vitro and in vivo experimental systems using CRISPR/dCas9-mediate editing have shown a strong effect on gene regulation bidirectionally as well as specificity. For example, dCas9-p300 and dCas9-dMSK1 induce an increase of target gene transcription through acetylation and phosphorylation to specific residues on histones, respectively [111, 112] (Table 2). Conversely, dCas9-HDAC1/3 silences gene expression by deacetylation of histone residues to induce compact chromatin status [113, 114]. Activity of dCas9 fused with histone methyltransferase or demethylase is dependent on cell type and developmental stages [115, 116]. As summarized in Table 2, dCas9-histone methyltransferase usually leads to gene silencing with other repressor components. A few of those enzymes show bi-directional regulations that depend on gene loci and associated molecular context [117, 118]. Thus, it is necessary to understand specific mechanisms in tissues or cell lines that are associated with imprinted impression when designing translational applications.

**Table 2:** Application of CRISPR-mediated epigenome editing.

Type	Function	Application	Reference
Chromatin remodeling			
dCas9-DNMT1 dCas9-DNMT3A dCas9-DNMT3B dCas9-DNMT3L dCas9-MQ1	DNA methyltransferase	Transcriptional repression	[92, 101–105]
dCas9-TET1CD dCas9-TET3CD	5-methylcytosine dioxygenase (DNA demethylation)	Transcriptional activation	[92, 107–109]
dCas9-VP64 dCas9-VPR (VP64-p65-Rta) dCas9-SunTag	Recruiting active transcription machinery Transcriptional activator/ Recruiting active transcription machinery Transcriptional activator	Transcriptional activation	[124, 125, 144]
dCas9-KRAB dCas9-KRAB-MeCP2	Transcriptional repressor	Transcriptional repression	[115, 128]
dCas9-HP1α	Heterochromatin binding protein	Chromatin compaction	[129]
dCas9-Ezh2 dCas9-G9a dCas9-SUV39H1	H3K27 methyltransferases H3K9 mono-, dimethyltransferase H3K9 trimethyltransferase	Transcriptional repression	[103, 115, 145]
dCas9-LSD1 dCas9-JMJD2A	H3K4me1/2, H3K9me1/2 demethylase H3K9me2/3, H3K36me2/3 demethylase	Transcriptional activation	[116, 146]
dCas9 SunTag-JARID1A	H3K4me3 demethylase	Transcriptional repression	[147]
dCas9-p300	Histone acetyltransferase	Transcriptional activation	[111]
dCas9-HDAC1 dCas9-HDAC3	Histone deacetylase	Transcriptional repression	[113, 114]
dCas9-dMSK1	H3S28 phosphorylase	Transcriptional activation	[112]
RNA modification			
dCas13-METTL3, /-METTL3METTL14, dCasRx-METTL3	N6-adenosine-methyltransferase	RNA stabilization, Translation efficiency	[89, 148, 149]
dCasRx-ALKBH5	m^6^A demethylase	RNA metabolism	[89]
RNA interference			
dCas13b-ADAR2	Adenosine deaminase acting on RNA	RNA editing for A to I	[83]
Cas13	RNA-guided RNA targeting	RNA knockdown	[76, 85]

To minimize off-target events and enhance precision, an inducible transient expression system has been introduced for epigenome editing [119–121]. Inducible dCas9 expression or activation allows tracking of its spatiotemporal control and potentially minimize off-target events because of the transient expression of dCas9. Technically, dCas9 fusion proteins are limited by their sizes over the maximal cargo size of AAV, a common and popular delivery tool. Novel delivery tools are necessary for in vivo application and clinical trials in humans. Alternative non-viral tools such as RNP or nanoparticle mediated delivery have emerged as better platforms, which have no limitations of package size [122, 123]. However, the molecular weight of current RNP or nanoparticle designs are too big to penetrate the brain efficiently. The cell type specificity of RNP and nanoparticle mediated delivery is poorly understood and this may limit clinical applications.

## Multiplexed CRISPRi and CRISPRa system

CRISPR/dCas9 mediated transcriptional interference (CRISPRi) or transcriptional activation (CRISPRa) systems have been actively developed due to eagerness for efficacy improvement. Various advanced versions of these systems can recruit multiple transcription factors to control gene transcription. More than two kinds of catalytic domains are fused with dCas9 for synergetic effects on target gene regulation [91, 96]. Beyond the introduction of four copies of herpes simplex viral protein 16 (VP64) as an activator, fusion or recruitment of multiple transcriptional factors have shown improved efficiency when manipulating target gene loci [124, 125] (Table 2). With dCas9 development, there have been trials for using combined or multiple gRNAs for more dramatic potency in target gene expression [126].

For epigenetic gene silencing, dCas9 is fused with Krüppel associated box (KRAB), derived from zinc finger domain, leading to decrease of chromatin accessibility and high H3K9me3 levels on target regulatory regions with recruitment of other repressors [127]. An advanced version of CRISPRi, dCas9-KRAB-MeCP2, has been shown to be highly effective as a transcriptional repressor [128]. While most dCas9 fusion proteins have cis-regulatory features, dCas9-HP1α acts in *cis* and *trans* by tethering to reach distal regulatory elements [129]. This tool also reduces chromatin accessibility by transcription machineries maintaining compact chromatin status.

## Illustrated examples of epigenetic therapy strategies for AS and PWS

**Fig. 2:**
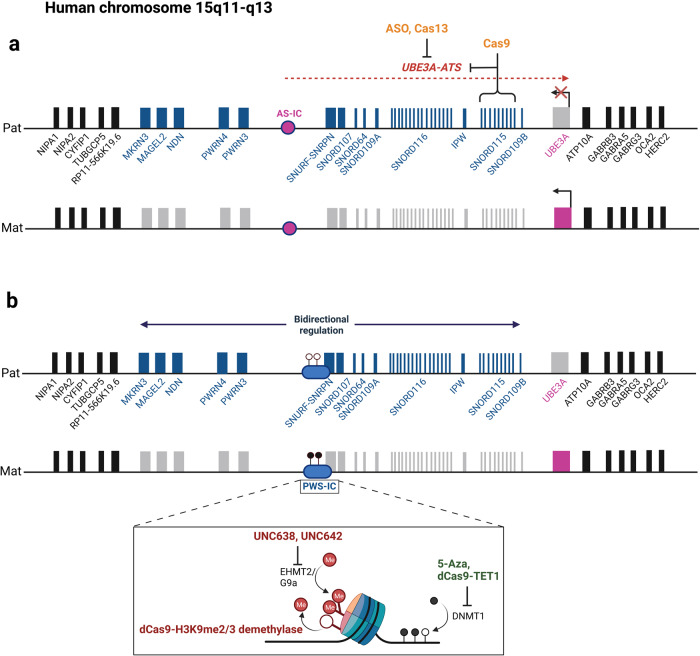
Therapeutic strategies for AS and PWS. Schematic shows the imprinting domain in human chromosome 15q11-q13 with potential epigenetic therapeutic candidates for (**a**) AS and (**b**) PWS. Genes in dark blue are exclusively expressed from the paternal chromosome while genes in purple are expressed from the maternal chromosome in neuronal cell type specific manner (gray bar, imprinted gene; biallelic expressed gene, black bar). In the case of AS, the loss of UBE3A expression in the maternal allele by different mechanisms is the cause. The principle of epigenetics-based therapy is to reactivate the paternal allele’s expression of UBE3A in neurons. The current approach is to inhibit the expression of antisense of *UBE3A* via small molecule, ASO, CRISPR/Cas9, or Cas13. In the case of PWS, where more than one paternally expressed gene is in the candidate region, the optimal approach is to manipulate the imprinting center region to reactivate the expression of silenced genes from the maternal chromosome. Current approaches include DNA methylation inhibitor, small molecule for histone modifications and others, CRISPR/dCas9 gene editing. Created with BioRender.com.

AS and PWS are caused by deficiency of maternally and paternally expressed genes on chromosome 15q11-q13, respectively. De novo ~6 Mb paternal deletion of the 15q11-q13 region is found in ~70% of PWS patients, followed by maternal UPD (~27%), and rare imprinting center mutations (Table 3) [130]. There are a dozen paternally expressed genes within the 15q11-q13 region (Fig. 2). *SNORD116* is considered to be the critical gene, where a deficiency is responsible for key PWS clinical features [21, 131]. The allele-specific expression of paternally expressed genes in the 15q11-q13 region is controlled by an imprinting center (PWS-IC) located upstream of the *SNURF-SNRPN* gene [132, 133]. The CpG islands located in the PWS-IC are unmethylated in the paternal chromosome but methylated in the maternal chromosome. Allele-specific histone modifications such as H3K9me2 are also associated with PWS-IC [60, 134]. These unique epigenetic defects render an opportunity to explore chromatin remodeling enzymes as therapeutic targets [21, 22]. Treatment with a DNA methylation inhibitor can reactivate the expression of *SNRPN* from the maternal chromosome in cells derived from a patient with PWS due to a paternal deletion of 15q11-q13 [135, 136]. In preclinical studies, treatment of EHMT2/G9a inhibitors have shown promise as potential pharmacological small molecules by reducing H3K9me2 levels, leading to reactivation of the imprinted gene without change of DNA methylation in a PWS mouse model [60]. Similarly, a fusion construct of dCas9 with H3K9me2/3 demethylase may be able to reduce the histone H3K9 methylation level at PWS-IC, resulting in reactivation of imprinted genes on the maternal chromosome. dCas9-TET1 could demethylate the *Snrpn* promoter region including CpG islands, suggesting a potent alternative tool [92]. Further preclinical studies are warranted to explore the therapeutic potential in human PWS.

**Table 3:** Molecular basis of Prader–Willi syndrome (PWS)/Angelman syndrome (AS).

Chromosome location	Imprinting disorder	Molecular defects (frequency)	Mouse model	Reference
15q11–q13	PWS	6MB paternal deletion (60%) mat UPD (36%) Imprinting defect (3%) Deletion of SNORD116 (rare)	pat-Del(Snrpn-Ube3a) 500 kb Deletion from Snrpn to Ube3a PWS-IC deletions of 4.8 kb/6Kb/35 kb	[21, 22, 150–155]
15q11–q13	AS	6MB maternal deletion (75%) pat UPD (1-2%) Imprinting defect (1–3%) UBE3A point mutation (5-10%)	mat-Del(7Gabrb3-Ube3a)^1yhj^ 1.68 Mb deletion from Gabrb3 and Ube3a Ube3a^tm1Alb^, Ube3a^Stop/+^, Ube3a^tm1Yelg^, Ube3a^tm2Yelg^ Ube3a Knockout	[18, 156–159]

In the case of AS, most patients (70%) have a de novo ~6 Mb maternal deletion including the *UBE3A* gene. About 10% have mutations within the *UBE3A* gene, followed by paternal uniparental disomy of chromosome 15 and imprinting center mutations [137–139]. *UBE3A* is the only maternally expressed gene within the chromosome 15q11-q13 region [69]. The expression of the maternal *UBE3A* allele is brain or neuron specific (Table 3) [70, 71, 140]. Intriguingly, there is no allele-specific epigenetic modification associated with AS-IC. The repressed expression of *Ube3a* in the paternal chromosome is mediated by a paternally expressed long non-coding RNA from the upstream region of *Snrpn* [18, 72]. The development of epigenetic and molecular therapy for AS has significantly advanced over the last decade [141]. Topotecan was the first drug showing a robust reactivation of *Ube3a* in a preclinical study in AS mouse model [59]. However, its translational potential is limited because of significant toxicity. The development of a safer new class of topoisomerase inhibitors remains a promising molecular therapy for AS. Treatment with topotecan can reduce the expression of *UBE3A-ATS* and reactivate the expression of *UBE3A* in human derived neurons and mouse neurons [59, 142]. Both ASO and Cas9 editing of *Ube3a-ATS* unsilence the paternal *Ube3a* allele by reducing *Ube3a-ATS* in AS mouse model [73–75, 143]. The success of ongoing ASO clinical trials will certainly promote translational studies exploring the use of Cas9 editing in human AS. The application of CRISPR/Cas13 mediated gRNA targeting *UBE3A-ATS* has been shown to be effective in AS mouse model [76]. Additional studies are warranted before advancing to IND studies.

## Concluding remark: the promises and challenges

With the FDA approved ASO in phase 1/2a trial for AS, the prospect of developing epigenetic molecular therapies for other genomic imprinting disorders is encouraging. The number of genomic imprinting disorders and the populations affected are relatively small. However, because of the unique molecular defects associated with genomic imprinting disorders and molecular mechanisms underlying imprinting regulations, genomic imprinting disorders remain the best opportunity for a proof of principle study of developing epigenetic therapy for genetic diseases. The lessons learned and tools developed are immediately applicable to other non-imprinting genetic disorders. For example, genetic defects that lead to haploinsufficiency of single genes are found in 10–15% of cases with ASD and even higher in other NDD. The approach of upregulating the expression of normal alleles by epigenome editing is an attractive avenue for developing molecular treatments. Despite these promises, challenges remain significant. For pharmacological based epigenetic therapy, the broad effects of epigenetic modulating drugs remain a potential concern for clinical applications particularly for mild or moderate presentation of the disease. It is possible that side effects are dose dependent and likely cell and tissue type specific. The careful assessment of these issues may ease the concern from FDA regulatory concerns. For ASO and gene editing based therapies, the efficiency of delivery platforms remains to be improved especially for the brain as a target organ. For example, the repeat dosing for ASO intrathecal delivery used in AS clinical trials is not optimal for patient care. The virus delivery platform for Cas9 editing has its own inherited and well recognized limitations. New non-viral delivery platforms have been developed in recent years but they are suboptimal for brain disorders. Lastly, for any gene editing approach, the genome wide off-target events of Cas9/gRNA remain a concern conceptually. The challenge is to assess the impact of off-target events technically and physiologically, and how to define an acceptable threshold of genome wide off-target events from a regulatory and safety perspective. Nevertheless, it is reasonable to predict that more promising breakthroughs utilizing CRISPR based genome and epigenome editing will emerge as an effective treatment modality in the near future.
